# On Phthisis and Other Diseases of Lima

**Published:** 1848-10

**Authors:** Archibald Smith


### *On Phthisis and other Diseases of Lima.* By Archibald Smith, M. D.

[For permission to copy the following interesting letter, (which, if not intended for publication by the author, is eminently worthy of it,) we are indebted to the kindness of Professor Dunglison. It gives a very different account of the liability to phthisis in Lima from what we have previously had; and furnishes additional proof, that mere uniformity of temperature of the most genial character does not afford that immunity from disease of the respiratory organs that we are accustomed to expect.—Ed.]

Robley Dunglison, M. D., Jefferson College, Philadelphia:

*Lima, 13th May,* 1848.

*Dear Sir,*—It has been my good fortune, a few days ago, to have met with a recent edition of the Cyclopædia of Practical Medicine, revised with additions by you. In turning to the article Climate, of this admirable work, I was surprised to find it stated, at p. 449, vol. i., that "Dr. Burrough resided four years in Lima, and did not meet with a single unequivocal case of phthisis which originated there during that period; although scrofula was not uncommon." Desirous to rectify the conclusions that may be drawn from this statement, put forth in a work of such high authority, and so ably revised as it is by you, I take the liberty to address to you this letter, in order that you should be put in possession of the *real fact*, that cases of *unequivocal phthisis* are not uncommon in Lima. How this prevalent disease could have escaped the observation of Dr. Burrough I cannot imagine, except it may have been that his practice was chiefly confined to foreign residents.

I flatter myself that you will kindly forgive this liberty, persuaded that the zeal and talent with which you successfully labour to diffuse the facts and reasonings of that profession of which I have the honour to be a member, will not be offended by any contribution which I can offer regarding the climate or diseases of Lima, with which a long residence has rendered me familiar in every class and rank of its population.

This capital covers a great extent of surface, and is interspersed with extensive orchards and convent grounds, all well watered by aqueducts, but productive of malaria, which at certain seasons—especially the autumnal—spreads ague in every street. The lower part of the town is only two leagues from the sea, and but a few hundred feet above its level whilst the higher part is not quite a league from the nearest chain of mountains; and the suburbs are detached by the river Rimac, over which there is a bridge of communication, and flanked by the adjacent hills of San Cristoval and Amencaes. Lima, thus situated between mountain and sea, and refreshed by a river, is free from the extremes of heat or cold. The temperature of the higher part of the city, which is skirted with orchards, is a few degrees cooler than that of the centre, near the cathedral and palace square. In the latter locality, during the dry season, the thermometer of Fahr. rarely rises above 80°—82°, and in winter it seldom falls below 60° within doors. The ordinary difference of range between the night and day thermometer is from three to four degrees in airy apartments, with brick floors, open windows and verandas, such as are common in Lima and on the coast. The hygrometer—Leslie's—seldom exceeds 50 degrees in the dry season—from November to May—or descends below 12 to 15 degrees in the cooler and more humid wintry months—from May to October. The barometer may be specified within the narrow range $29\frac{9}{10}$ to $29\frac{1}{2}$. Rain is so rare as to be scarcely ever witnessed; for what is called the wet season in Lima and its environs is little more than a morning and evening haze, with a kind of Scotch mist or drizzle. The atmosphere of the coast appears to have little or no electricity. Thunder and lightning are, like hail, snow and sleet, unknown in this bland atmosphere: on the Cordillera, indeed, these wars of the elements are prevalent during the wet season, when it rains in torrents; but even in these Alpine regions it seldom rains before 2 P. M. On the Andes or Cordilleras, as on the coast, the seasons are properly divided into the wet and the dry. But the dry months of the mountains are the wet of the coast, and the wet season of the coast the dry on the mountains. On the coast, January, February, and March are the hottest of the dry months; and June, July, and August the most foggy and drizzly of the wet season.

The inhabitants of Lima and its adjacent maritime valleys are, many of them, of white parentage, but more generally a mixed race of all grades between the Indian and African, as well as between the white and Indian, and the white and negro. The white infant is generally reared at the breast of the black nurse; and is frequently passed from one to another, until the parents think that they have secured a proper one; but with all the care and anxiety inseparable from such transitions, the white child is often tainted in its blood from its earliest infancy. The white mother is, in this fair, but deceitful, climate, so prone to *hæmoptysis and phthisis as a consequence of nursing*, that she is hardly ever able to suckle with impunity. But though in this respect the white parent is more tender than other women of inferior caste, yet delicacy of constitution is not confined to females of any class. Among all ranks of the community a general laxity and weakness of habit is prevalent. Errors of diet and education, with the entire neglect of medical police, may have much to do in developing the weakly physical character of these people; yet independently of all such causes, there is a want of tone or energy inseparable from the climate itself, which affects not only man, but also, in a most remarkable degree, the dog species, soon depriving even the English bull-dog of his ferocity and vigour. In short, whatever the cause may be, the fact cannot be concealed, that the European soon degenerates in this climate; and that the native, whether of European or African descent, shows an early predisposition, even in childhood, to habitual congestive diseases, such as hæmorrhoids; blenorrhœa; uterine, intestinal and pulmonary catarrh, which are too often precursory of dysenteries, cancer uteri, phthisis, asthma with disease of the heart and dropsy; not to mention gastric and other fevers consequent on a deranged state of the stomach, liver and bowels, besides the aggravating effects of the prevailing *malaria,* which always displays its influence when the general health is impaired by divers other diseases. In the autumnal season especially, convalescents from other acute diseases are very prone to ague in some form or other. Yet in spite of all such multifarious ills, the climate of Lima has ever been exempt from any great or sweepingly fatal epidemics. Typhoid symptoms are sometimes sporadic in certain cases of fever, but never contagious; and the yellow fever has never approached nearer than Guayaquil, on the equator—12 degrees north of Lima.

With regard to phthisis, when imported by sickly sojourners from Chili and other parts, it is true that such invalids seem to get better for a short time after their arrival in Lima; but the amendment is more in the feelings of the patient, soothed for a while by the comparative mildness of the air, and the out-door exercise which animates the sinking spirits, after the confinement and monotony of a sea voyage of greater or less duration. But the painful truth remains to be told, that though the organic lesions may thus be more or less modified, or arrested, for a time, especially during the summer months, yet the disease, in the long run, makes sure progress to its fatal termination. It is usual, in consultations on such exotic cases, for Lima physicians to recommend the patients to retire from the capital with the least possible delay.

Hæmoptysis is often symptomatic of simple pulmonary congestion in the fevers of the coast—especially among negroes—and of amenorrhœa among young females. But be its origin what it may, its appearance is always associated with the idea of phthisis; because most of the fatal cases of this destructive disease in Lima are attended with spitting of blood at some stage of their progress: hæmoptysis—one of the most prevalent disorders in Lima—is therefore looked upon by every Limenian as a precursory symptom of phthisis pulmonalis; and its fatal consequences are so well known to the physician, and so dreaded by all, that the patient who hopes to escape with his life is ordered to leave the warm and humid air of the coast for the more congenial air of some inland valley of the Andes, where every diversity and gradation of temperature may be met with. The very warm and dry atmosphere of some inland valleys does not appear the most suitable, even though the *annual range* of the thermometer (Fahr.) be only from 66° to 72°, as, with little variation, is the case in the vale of Huanuco, between the Eastern and Western Cordilleras. Tarma and Canta, both about 10,000 feet above the level of the sea, and of a moderately humid temperature, at about 60° Fahr., are the favourite and approved haunts of hæmoptoic convalescents from Lima. Scrofula, in form of what the natives call "lamparones," or clusters of swelled glands in the neck, is a common disease among all castes, but more especially in a certain dark race of mulatto and negro parentage, very much employed as domestics, and from whose number nurses for the white children are often provided; though, when it can be done, the negress is always chosen, as it is imagined that her milk is less heating. The black cow's milk is also preferred on the same idea. By popular consent and phraseology all things eatable or drinkable are divided into cold and hot; but in the application of this fundamental principle of Spanish-American dietetics, very fatal vulgar errors are committed; and particularly in cases of incipient pulmonary disease, wherein they attribute all to "*ardor interior*," or an internal heat, which they offer to correct by *iced beverages*, sitting in draughts between open doors and windows, and exposing the chest and shoulders to the chilling action of such artificial currents of air—very often the cool night air!

In order to show the extent of mortality in Lima, and the relative proportion of fatal diseases, I shall subjoin an authentic Table of Mortality, from the official reports of the public cemetery or Pantheon of Lima, during the five years 1842 to 1846. This Table, however, does not include English or Americans *not* Roman Catholics, who have a burying place of their own near the seaport of Callao—two leagues from the capital.

It may be interesting to premise that the whole population of Lima, by the latest census made about ten years ago, did not quite reach 56,000; but that, allowing for the great influx of foreigners, and even native families from remote provinces, during the last ten or twelve years, the present number may be computed at 60,000, or thereabouts, which it is well to bear in mind when considering the great annual mortality in the Table.

| Diseases. | Total of the Five Years. | Average of. |
|---|---|---|
| Dysentery, | 2381 | $476\frac{1}{5}$ |
| Fevers, | 2547 | $509\frac{2}{5}$ |
| Tabardillo or low nervous fever, | 363 | $72\frac{3}{5}$ |
| Phthisis, | 1548 | $309\frac{3}{5}$ |
| Pleurisy and pneumonia, | 774 | $154\frac{4}{5}$ |
| Dropsy, | 520 | |
| Cholera morbus, | 8 | $1\frac{3}{5}$ |
| Malignant pustule, | 2 | $\frac{2}{5}$ |
| Small pox, | 118 | $23\frac{3}{5}$ |
| Convulsive cough, | 22 | $4\frac{2}{8}$ |
| Sudden deaths, | 206 | $41\frac{1}{5}$ |
| "Fusilados" or shot, | 11 | $2\frac{1}{5}$ |
| Various diseases, | 7280 | 1456 |
| | 15780 | 3156* |

*Remarks.*—Suppose the population of Lima 60,000, and the deaths in the same 3156 yearly, it appears that in less than twenty years a number equal to that of the whole population is carried off by ordinary diseases.

The great amount of deaths under "Various Diseases" not only includes a large proportion of fatal infantile diseases—especially dentition—which occur in private families, but also of the poor who die in hospital, and whose diseases are not regularly specified in the returns of the Administrator of the Public Cemetery, as are those of such as die in private houses, and have to be duly reported by the parochial curates; who, for special reasons, are very attentive to the discharge of this duty.

Now, in reference to consumption or phthisis pulmonalis, (so prevalent among the poor in hospital during the spring and autumn months,) were the fatal cases distinctly recorded instead of being indiscriminately huddled under the head of "*Various Diseases*," the mortality from phthisis would be seen to exceed very considerably the numbers stated in the above Table of Lima Mortality. And it is of great moment to state explicitly in this place, that of at least thirty-eight or forty dissections lately made in hospital of those patients who have died with the ordinary symptoms of phthisis—(such as cough, purulent expectoration, and

* The year 1847 exceeded this average: the deaths were 3400—small pox prevalent. [Some interesting observations on the same subject are contained in Tschudi's "Travels in Peru during the Years 1838—1842." New York, 1847. Ed.]

colliquative sweats or diarrhœas, with hectic fever, &c.) all have, on inspection, presented the lungs in a highly tuberculated condition—*in* some instances all over studded with tubercles.

Under the head "Convulsive Cough" are included suffocative catarrh and croup. The latter is not a common disease; but last week I attended, in consultation, a case of angina membranacea, which was complicated with secondary croup, clogging the air tubes by ropy mucous secretion, and false membranes, of which pieces were ejected by coughing. It proved fatal on the eighteenth day from the invasion of the angina. During the hot months, when the irritability of the whole system is greatly increased on the coast, the mucous membrane of the stomach and bowels becomes readily affected, and cholera morbus is a common result; but as this disease is almost always cured with *ice*, the deaths, as above, are few.

For a further account of Peru and its diseases permit me to refer to "Peru as it is," published by Bentley, London, 1839, in which I would particularly refer to the Chapter on Limenian Dietetics, in Vol. I.; and likewise to what I have written, entitled "Practical Observations on the Diseases of Peru, described as they occur on the coast, and in the Sierra," in the Edin. Med. and Surg. Journal, in successive papers—to be seen in No. 143, 144, 145, 148, 149, 151, 152. And asking your indulgence for so long and crammed a letter,

With the highest consideration and respect,

Allow me to be your very obedient servant

And well wisher,

ARCHIBALD SMITH.

